# Tick-Borne Viruses in a Changing Climate: The Expanding Threat in Africa and Beyond

**DOI:** 10.3390/microorganisms13071509

**Published:** 2025-06-28

**Authors:** Cara Leonie Ebert, Stefanie C. Becker

**Affiliations:** 1 Research Group for Vector-Associated Biodiversity and Infection, Buenteweg 17, 30559 Hanover, Germany; cara.leonie.ebert@tiho-hannover.de; 2 Research Center for Emerging Infections and Zoonoses, Buenteweg 17, 30559 Hanover, Germany

**Keywords:** tick-borne viruses, Crimean–Congo hemorrhagic fever virus, climate change, Africa

## Abstract

Tick-borne viruses (TBVs), notably *Orthonairovirus haemorrhagiae* (Crimean–Congo hemorrhagic fever virus, CCHFV), are emerging global health threats intensified by climate change. Rising temperatures and altered precipitation patterns are expanding the habitats of key tick vectors, increasing their survival and reproductive success. The African continent is characterized by many different climatic zones, and climatic shifts have increased or changed CCHFV transmission patterns, becoming greater risk to humans and livestock. Beyond Africa, CCHFV spread in Europe, the Middle East, and Asia and has been facilitated by factors such as livestock movement, deforestation, and migratory birds. Climate-driven shifts in tick seasonality, behavior, and vector competence may further enhance viral transmission. Addressing these challenges requires integrated responses, including enhanced surveillance, predictive modeling, and climate-adaptive vector control strategies. A One Health approach—linking environmental, animal, and human health domains—is essential. Innovative strategies such as anti-tick vaccines and sustainable vector control methods offer promise in reducing the burden of these diseases. Proactive, collaborative efforts at regional and international levels are crucial in tackling this growing public health challenge.

## 1. Introduction

TBVs are increasingly recognized as significant public health threats, with climate change acting as a major driver of their global emergence and re-emergence [1]. Among these viruses, CCHFV poses a particular concern due to its expanding range and severe public health implications [2]. CCHFV is primarily transmitted by *Hyalomma* ticks and has been recognized by the World Health Organization as a high-priority pathogen due to its epidemic potential and the lack of effective treatments or vaccines [3,4]. Recent studies highlight the strong influence of climatic variables—particularly rising temperatures and changes in precipitation patterns—on the spread of the disease. For instance, statistical analyses have revealed significant associations between increasing annual temperatures and CCHF (Crimean–Congo hemorrhagic fever) incidence, especially in regions such as Iran, Turkey, and Russia, where warming trends have contributed to the geographical expansion of tick habitats and virus transmission cycles [5].

Furthermore, land-use changes and ecological shifts, often driven by climate change, have increased the risk of human exposure to infected vectors and hosts, exacerbating the emergence of new foci in both endemic and non-endemic regions [6].

The growing distribution of ticks, particularly *Hyalomma* and *Rhipicephalus* species, is closely linked to rising temperatures, shifting precipitation patterns, and land-use changes that create more favorable conditions for tick survival and reproduction [7,8,9].

Africa, a continent already burdened with high rates of vector-borne diseases, is witnessing a climate-driven shift in tick habitats, with some species expanding their range while others retreat to new ecological niches [10,11]. The interplay between climate change, human activity, and ecosystem alterations is accelerating the spread of tick-borne pathogens. Among these pathogens is CCHFV, which poses a threat to both human and animal populations [12]. The rise in livestock trade, deforestation, and migratory bird movements further complicates this scenario, facilitating the introduction of new tick species and their associated pathogens into previously unaffected regions [7,13,14].

Climate change also influences tick-borne disease seasonality and vector competence, impacting how efficiently ticks transmit viruses to their hosts [15]. Unlike mosquitoes, whose populations fluctuate rapidly with changing weather conditions, ticks develop more slowly and respond more gradually to environmental shifts [16]. As a result, the impact of climate change on TBVs is expected to progress incrementally, underscoring the need for proactive and long-term monitoring efforts. Additionally, shifts in tick microbiomes and immune mechanisms due to environmental stressors could further influence their ability to transmit viruses [17,18].

The spread of CCHFV beyond Africa into parts of Europe, the Middle East, and Asia raises concerns about global health security, with new cases emerging in regions where the virus was previously absent [19,20]. This expansion highlights the need for integrated One Health strategies, combining climate modeling, ecological surveillance, and public health preparedness to mitigate emerging threats. Novel approaches, such as anti-tick vaccines or microbiome manipulation, are essential in limiting the growing burden of tick-borne diseases [21].

As climate patterns continue to shift, the urgency of international collaboration in tick-borne disease monitoring and control cannot be overstated. To fight tick-borne diseases, we must understand how ticks and viruses interact. This includes their ecology, immune responses, and behavior. Such knowledge will improve models, diagnostics, and control methods [22,23]. This paper explores the expanding risk of CCHFV and other TBVs in Africa and beyond, emphasizing the role of climate change in shaping future disease landscapes.

## 2. Major Tick-Borne Viruses Affecting Africa: CCHFV, NSDV, and ASFV

Several TBVs of high veterinary and public health importance are endemic to the African continent. Among these, CCHFV, *Orthonairovirus nairobiense* (Nairobi sheep disease orthonairovirus, NSDV), and African swine fever virus (ASFV) represent distinct threats due to their zoonotic potential, high mortality rates, and expanding geographic ranges.

### 2.1. Orthonairovirus Haemorrhagiae (CCHFV)

CCHFV is a negative-sense, single-stranded RNA virus of the genus *Orthonairovirus* (family *Nairoviridae*, class *Bunyaviricetes*), responsible for CCHF in humans. The disease is characterized by the sudden onset of fever, muscle pain, blood-clotting disorders, and, in severe cases, hemorrhagic manifestations, with case–fatality rates exceeding 30% [4,24]. The virus is primarily transmitted by *Hyalomma* spp. ticks, which serve both as vectors and reservoirs. Infected domestic animals—such as cattle, sheep, and goats—typically remain asymptomatic but can carry high viral loads and contribute to human exposure through occupational contact or tick bites [25,26].

Wild vertebrates, including hares and certain rodents, may also be involved in the enzootic maintenance cycle [27]. Migratory birds are not susceptible to CCHFV replication; however, they play a crucial role in the geographic dissemination of infected *Hyalomma* ticks [25]. This mechanism is suspected to contribute to the emergence of CCHFV in new areas of Europe, the Middle East, and Central Asia, particularly under the influence of climate-induced changes in bird migration routes and tick phenology [28,29].

### 2.2. Orthonairovirus Nairobiense (NSDV)

NSDV is closely related to CCHFV and is also referred to as Ganjam virus in South Asia. It causes a highly lethal hemorrhagic gastroenteritis in sheep and goats, with mortality rates of up to 90% in naïve populations. Transmission primarily occurs via *Rhipicephalus appendiculatus* and *Amblyomma* spp. ticks, with East Africa being the core endemic region [30,31]. Furthermore, evidence suggests that NSDV spreads passively due to migratory birds carrying tick vector species [32]. Similarly to CCHFV, NSDV is also assumed to be able to spread through migratory birds and animal trade, which could have severe effects on food supplies [31,32].

### 2.3. African Swine Fever Virus (ASFV)

ASFV is a large, enveloped, double-stranded DNA virus of the family *Asfarviridae*, representing the only known DNA arbovirus [33,34].

It causes African swine fever (ASF), a hemorrhagic disease in domestic and wild suids with mortality rates approaching 100% in susceptible pig populations [35]. ASFV is primarily transmitted via direct contact, the ingestion of contaminated material, or through *Ornithodoros* spp. soft ticks, particularly *Ornithodoros* (*O.*) *moubata* in sub-Saharan Africa. The sylvatic cycle involves asymptomatically infected warthogs and soft ticks, which maintain viral circulation within burrows and natural habitats [36].

Once introduced into domestic settings, ASFV spreads rapidly, especially under conditions of inadequate biosecurity [37]. The virus spread from Africa to Europe and Asia in the last century and is found in native wild boar populations. From here, it can also spread directly to domesticated pigs, leading to severe outbreaks and the culling of pigs to prevent further spread [38,39].

## 3. Climate Change and Tick Distribution in Africa

**Figure 1:**
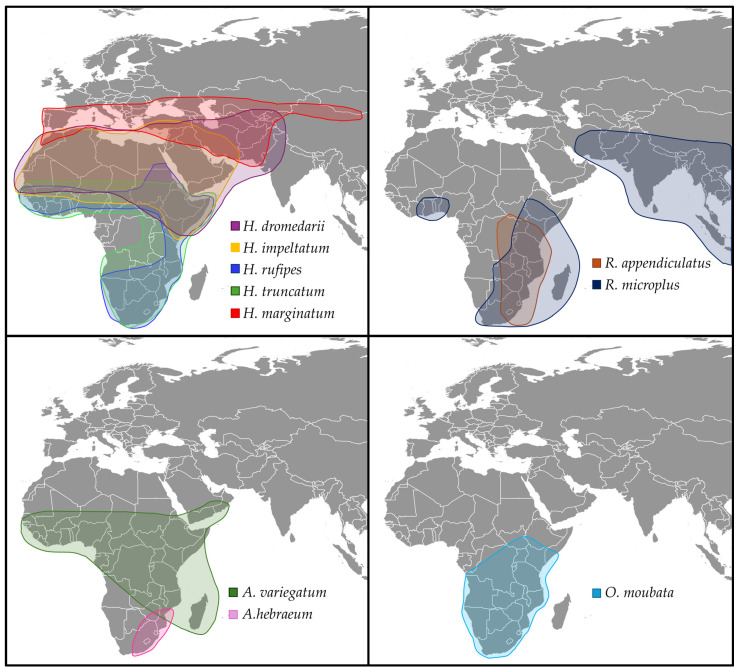
**Geographic distribution of the most relevant tick species in Africa of importance for human and animal health.** Shown are the known distribution ranges across Africa, Europe, and Asia, based on published occurrence data [49,73,74,75,76].

Climate change is expected to have a profound impact on the distribution of ticks in Africa, with consequences for both animal and human health. However, the exact extent of this influence remains unpredictable due to the complex interplay among climate, biodiversity, tick biology, pathogens, human behavior, and ecological processes [40]. While climate, including temperature and humidity, is a major driver of tick distribution, other factors such as land-use changes, livestock movement, and human population growth also play critical roles in shaping the increase and spread of tick populations [10]. Figure 1 shows the distribution of the most relevant tick species of these genera, and Table 1 summarizes the distribution of tick species, as well as the impact of climate change on these tick species.

### 3.1. Relevant Tick Species from the African Continent

Ticks, arachnids from the class Acari, are obligate hematophagous ectoparasites that infest several vertebrate species, including humans and livestock. During the blood feeding process, ticks can transmit various pathogens, including viruses, bacteria, and protozoa [41]. The most prominent vectors for tick-borne pathogens are species from the family *Ixodidae* (hard ticks), which are distinguished by a rigid dorsal shield and are responsible for the majority of disease transmissions. However, ticks from the family *Argasidae* (soft ticks) also play an important role—particularly in arid habitats—where they can serve as significant vectors for pathogens affecting both humans and animals [42]. Due to its diverse ecological zones, the African continent has several relevant tick species, most significantly from the genera *Rhipicephalus*, *Amblyomma*, and *Hyalomma*, that pose a significant health burden [43].

Among the most prominent tick species in Africa are those belonging to the genus *Rhipicephalus*, which transmit a variety of pathogens, especially *Babesia* spp. Notably, *Rhipicephalus* (*R.*) *microplus* is an invasive and widespread tick species in tropical and sub-tropical regions that causes significant ecological damage in cattle [44].

*R. appendiculatus* serves as a principal vector of *Theileria parva*, the protozoan parasite responsible for East Coast fever in cattle [45]. This tick-borne disease contributes to substantial economic losses in the livestock sector, particularly across regions of Eastern and Southern Africa. *R. appendiculatus* is also the main vector of NSDV in eastern Africa [30,31].

Among ticks of the *Amblyomma* genus, which are predominantly found in southern Africa, the most veterinary-relevant species are *Amblyomma* (*A.*) *variegatum* and *A. hebraeum* [43]. For example, this species transmits *Ehrlichia ruminantium*, the etiological agent of heartwater disease. This condition affects ruminants such as sheep, goats, and cattle and can result in acute mortality if not promptly diagnosed and treated [46,47].

In arid and semi-arid zones, particularly in the Sahel and northern Africa, ticks belonging to the *Hyalomma* genus play a significant role as the main vectors of CCHFV [4,48]. In contrast to many other ixodid ticks, which typically engage in passive host-seeking behavior (questing), *Hyalomma* species exhibit active host pursuit [48]. In Africa, CCHFV has been detected in several *Hyalomma* species, e.g., *Hyalomma* (*H.*) *dromedarii*, *H. impeltatum*, *H. rufipes*, *H. truncatum*, or *H. marginatum* collected from domestic and wild animals such as cattle, camels, and antelopes across countries like Nigeria, Senegal, Ethiopia, Kenya, or South Africa. Furthermore, it has been demonstrated that these species are competent vectors for CCHFV [49,50,51,52,53,54].

Furthermore, *O. moubata*, a member of the *Argasidae* (soft ticks) and vector for ASFV, is frequently encountered in traditional dwellings throughout Eastern and Southern Africa. This species is a known vector of *Borrelia duttonii*, the causative agent of African relapsing fever in humans, a condition characterized by recurrent episodes of fever due to antigenic variation in the pathogen [55].

### 3.2. Shifts in Tick Habitats, Behavior, and Seasonal Disease Risks

Rising temperatures and changing precipitation patterns are altering habitat suitability for various tick species. For example, in South Africa, a temperature increase of 2 °C is predicted to reduce habitat suitability for *R. decoloratus*, *A. hebraeum*, *R. appendiculatus*, and *H. truncatum* [56]. Similarly, by 2050, the potential habitat of *A. hebraeum* in Mashonaland Central Province, Zimbabwe is expected to decrease by approximately 13%, with temperature, rainfall, and elevation being the key environmental drivers [57].

However, not all tick species are expected to decline. Back in 2007, Olwoch et al. used their climate model to show significant shifts in the distribution of 30 *Rhipicephalus* species across Africa, with East and Southern Africa being particularly vulnerable to these changes. Over 50% of species are projected to expand their range, with economically important tick species making up 70% of this expansion. Additionally, approximately 20% of tick species may shift their distribution range by 50–100%, leading to increased tick species richness in southwestern Africa [10]. In Northern Ethiopia, climatic changes are expected to expand the distribution of *Rhipicephalus* ticks into new areas, increasing the incidence of tick-borne diseases and negatively affecting cattle production [58]. Furthermore, the movement of livestock has facilitated the expansion of invasive tick species such as *R. microplus* and *A. variegatum*, both of which have been spreading in Africa, contributing to the emergence or re-emergence of tick-borne diseases [59]. Additionally, *R. microplus* has spread further across Tanzania, while *R. decoloratus* has largely retreated to highland areas, with their distributional boundary following a climate gradient [60].

Ticks respond differently to climate changes compared to other vectors such as mosquitoes due to differences in their life cycles. Mosquito populations can quickly adapt to short-term climate variations, whereas tick populations respond more gradually to long-term climate trends. Consequently, tick-borne disease risks are expected to evolve steadily over time rather than fluctuating annually [16].

Tick distribution is also strongly influenced by seasonal and ecological factors. For example, it has been shown that in Eastern Cape Province, South Africa, tick populations vary significantly across agroecological zones and seasons. For instance, *A. hebraeum* and *R. evertsi evertsi* were most common in Kowie Thicket during summer, while *R. appendiculatus* was present across multiple seasons [61]. Additionally, *H. truncatum* and *H. rufipes* exhibit distinct seasonal activity patterns, with larvae peaking in July and November, suggesting two generations per year. Adult *H. rufipes* is most abundant between September and March, while *H. truncatum* peaks slightly earlier [62].

Climate change also influences tick questing behavior, which could impact interactions with hosts and the transmission of tick-borne pathogens. Changes in tick questing behavior due to climate change may also impact the effectiveness of tick control efforts that target tick behavior [63].

### 3.3. Environmental Conditions Favoring Tick Expansion

Ticks thrive in warm and humid conditions, and changes in temperature and precipitation are altering their distribution. In general, it has been shown that factors such as minimum and maximum temperatures, along with rainfall, are strong predictors of tick distribution, rather than vegetation-related factors [64]. In addition, ticks are adaptable to certain environmental conditions, and it has been shown that tick populations of the same species may adjust to the particular climate of the region that they inhabit, indicating that the species as a whole may possess a certain resilience to climatic changes [65]. In addition to temperature and precipitation, other environmental factors significantly contribute to tick expansion across Africa. Land-use changes, such as deforestation and the expansion of agricultural areas and animal farming, lead to habitat changes, which facilitates increased contact between wild and domestic hosts, as well as direct tick–livestock contact, and thereby enhances opportunities for tick proliferation and pathogen transmission [66,67,68]. Additionally, ticks belonging to genera such as *Amblyomma*, *Haemaphysalis*, *Hyalomma*, *Ixodes*, and *Rhipicephalus* are generalists that parasitize a broad range of hosts, including both domestic and wild herbivores, as well as carnivores, further facilitating their survival in changing environments [69]. In South Africa, for example, tick infestations were highest in KwaZulu-Natal (45%), followed by Limpopo (26%) and Eastern Cape (19%). The most prevalent species included *A. hebraeum* (55.1%), *R. evertsi evertsi* (13.9%), and *R. decoloratus* (11.9%). Tick infestations were particularly high in areas with poor grazing practices, insufficient acaricidal treatment, and traditional animal husbandry [70]. These shifts, often associated with broader patterns in climate change, contribute to more stable tick populations and prolonged periods of seasonal activity, ultimately increasing the risk of infestation in both livestock and wildlife.

### 3.4. Future Research and Disease Control Strategies

It has been shown that Africa is experiencing long-term warming of 0.1–0.3 °C per decade, with significant rainfall variability, particularly in the Sahel and East African long rains [71]. While climate data and seasonal forecasts can help predict vector-borne disease risks, their accuracy remains variable.

For this, integrated “One Health” research is needed to monitor tick distribution changes, identify how these shifts are driven by climate change, and develop predictive models for public and animal health planning [1]. There is also a growing need for research into TBVs in sub-Saharan Africa, as 14 TBVs have been reported, 8 of which are zoonotic, with increased research interest emerging since 2021 [72].

**Table 1:** **Overview of key tick species in Africa and the projected impact of climate change on their distribution**. This table summarizes selected tick species of major veterinary and public health relevance across the African continent. It includes their genus, the expected impact of climate change on their habitat suitability and geographic distribution, and the regions most likely to be affected. Notes highlight specific ecological or epidemiological concerns, such as range expansion, species replacement, or climate sensitivity. References are provided for each data point.

Tick Species	Genus	Climate Impact	Region(s) Affected	Notes	References
***R. appendiculatus***	*Rhipicephalus*	Habitat suitability reduced with +2 °C warming. Expansion into new areas with shifting rainfall patterns.	South Africa, Northern Ethiopia	Vulnerable to rising temperatures; expanded range in some regions.	[56]
***R. microplus***	*Rhipicephalus*	Expanding range due to livestock movement and warming; replacing native *R. decoloratus*.	Tanzania, Cameroon, sub-Saharan Africa	Invasive; thrives in warmer lowlands.	[59,60,76]
***R. decoloratus***	*Rhipicephalus*	Retreating to highland areas with cooler temperatures.	Tanzania	Losing ground to *R. microplus*.	[60]
***A. hebraeum***	*Amblyomma*	Predicted 13% decrease in habitat by 2050 due to temperature and rainfall shifts.	Zimbabwe (Mashonaland Central), South Africa	Sensitive to climate variables.	[57]
***A. variegatum***	*Amblyomma*	Expanding due to animal movement and land use changes.	Multiple African countries	Climate-tolerant; generalist feeder.	[59]
***H. truncatum***	*Hyalomma*	Habitat suitability is decreasing in warming regions.	South Africa	Two larval peaks; impacted by temperature.	[62]
***H. rufipes***	*Hyalomma*	Seasonal activity altered; more generations per year possible.	Eastern Cape Province, South Africa	Adults peak Sept–March; larvae July–Nov.	[62]
***H. marginatum***	*Hyalomma*	Expanding into Europe due to warmer, drier summers and moderate autumn rains.	Spain, France (Var, Ardèche, Pyrénées-Orientales)	CCHFV risk in the Mediterranean Basin.	[65,77]
***O. moubata***	*Ornithodoros*	Expected to expand under warming; altered feeding rates and reproduction.	Eastern and Southern Africa	Soft tick; vector of ASFV and relapsing fever.	[55]

## 4. Impact of Climate Change on Tick-Borne Viral Transmission in Africa and Beyond

Climate change is increasingly recognized as a key driver of shifts in tick-borne viral transmission [1,78]. At a broader scale, the emergence of vector-borne diseases—predominantly associated with viruses from *Flaviviridae*, *Togaviridae*, and *Bunyaviricetes*—has intensified globally, with Africa exhibiting the highest incidence of such events after accounting for surveillance bias [79]. Rising temperatures, altered precipitation patterns, and changing ecosystems are affecting tick activity, distribution, and host interactions, ultimately influencing the spread of viruses such as CCHFV, ASFV, and NSDV [1,7,15,80].

However, tick-borne disease systems are highly complex, making it difficult to distinguish the specific effects of climate change from other contributing factors such as land-use changes, wildlife trade, and animal movement [81]. Tick species and the viruses transmitted by these species are summarized in Table 2.

### 4.1. The Role of Climate in Tick-Borne Virus Transmission

Climate change affects tick population dynamics, which in turn influences the transmission of TBVs. Temperature and humidity play key roles in tick survival, reproduction, and questing behavior, with heat stress responses such as the expression of heat shock proteins involved in regulating tick activity and pathogen transmission [15]. Elevated temperatures, for example, can lead to increased tick questing behavior, increasing the risk of disease transmission [82,83]. Notably, tick saliva plays a crucial role in virus transmission, as it contains antimicrobial protein. Understanding the ecological, immunological, and behavioral dynamics of tick–virus interactions will be crucial for developing predictive models, enhancing diagnostic capabilities, and implementing sustainable disease mitigation strategies and other components that facilitate virus–host interactions. Understanding these mechanisms could provide new targets for anti-tick vaccine development [84]. Moreover, different pathogens have evolved similar immune manipulation strategies to infect vectors and hosts, highlighting the potential for broad-spectrum interventions that disrupt these shared transmission mechanisms [18].

### 4.2. The Growing Threat of CCHFV

Climate change not only affects tick distribution but also influences the prevalence of tick-borne viruses such as CCHFV. Since 2000, 9 African countries have reported their first confirmed human CCHF cases, and between 1956 and 2020, at least 494 human cases were recorded across 19 African countries, with 115 fatalities [85]. In addition, the virus has been detected in *R. decoloratus* and other *Rhipicephalus* ticks collected from livestock markets and slaughterhouses in Kenya [86].

Regions with high human population density, large areas of grassland, and high shrub cover tend to have a greater risk of CCHF outbreaks, whereas increased precipitation, elevated mean temperature, and slope have been linked to lower outbreak risks. Therefore, the Sahel region of West Africa and southeastern East Africa have been identified as high-risk zones for CCHF outbreaks [87].

CCHFV is one of the most important TBVs in Africa, with a broad host range that includes wild and domestic animals. Wild animals serve as reservoirs, while livestock amplifies the virus, additionally increasing tick populations and consequently, human exposure risk. In addition, birds contribute to the virus’s spread across regions [25].

The spread of migratory birds, illegal wildlife trade, and livestock transport are major contributors to the increasing risk of CCHFV transmission [88]. In Uganda, a cross-sectional study of urban abattoirs receiving cattle from across the country found high CCHFV seropositivity in humans (10.3%) and cattle (69.7%), with CCHFV antigens primarily detected in *R. appendiculatus* ticks. Ecological modeling indicated a high human CCHF risk across much of Uganda, with low predicted suitability for *Hyalomma* ticks but high suitability for *Rhipicephalus* and *Amblyomma* ticks [89].

CCHFV mortality rates and prevalence are highest in low-income regions such as Africa, where tick control measures and healthcare infrastructure remain limited. While *Hyalomma* ticks are the primary vectors, *Rhipicephalus* and *Amblyomma* may contribute to transmission [90]. Furthermore, risk mapping indicates higher CCHF outbreak risks in semi-arid and savanna regions, where human exposure to infected ticks is more likely [87].

### 4.3. The Expanding Risk of CCHFV: From Africa to Emerging Hotspots in Europe

CCHFV is an emerging global health threat, historically endemic to Africa, Asia, the Middle East, and southeastern Europe. Over the last two decades, CCHFV and its tick vector have expanded into previously unaffected regions, including parts of southern and central Europe, largely driven by climate change, socioeconomic factors, and migratory bird patterns [19].

Across the Mediterranean basin, for instance, annual mean temperatures have increased by approximately 1.4 °C compared to the late 19th-century baseline [91].

This shift likely contributed to the recent emergence of CCHFV in Spain, where 17 cases were reported between 2013 and 2024, with six deaths [20]. Surveillance efforts have also identified *H. marginatum* populations in multiple regions of France, including the Pyrénées-Orientales, Var, and Ardèche, where clustered distributions indicate ongoing colonization. It has been shown that the tick’s presence is strongly associated with warm temperatures, dry summers, and moderate autumn precipitation [77]. Temperature and rainfall patterns are predicted to continue to influence Mediterranean regions such as southern France and Spain, favoring the further establishment of vector-borne diseases such as CCHF [91]. Moreover, spatial modeling suggests that CCHF could spread further into Southern, Central, and Western Europe, with the Mediterranean region being particularly suitable for *Hyalomma* tick establishment. Some models indicate a northward expansion, likely influenced by climate change [92].

### 4.4. Climate Change and the Role of Migratory Birds in CCHFV Spread

The continued spread of *H. marginatum*, the primary tick vector to Europe, raises concerns about CCHFV emergence in new areas, necessitating updated predictive models and surveillance to assess future risks [93]. Migratory birds are important in the geographic expansion of *Hyalomma* ticks, potentially introducing CCHFV to new regions. However, a model from 2012 predicted that while rising temperatures may increase tick development and molting rates, they may reduce tick abundance on birds, ultimately making the climate-driven risk of CCHFV spread to Europe via migratory birds low [94]. Despite this, migratory patterns remain a concern, especially in coastal and Mediterranean regions where favorable conditions for tick establishment exist.

### 4.5. Assessing the Risk of CCHFV Introduction in CCHF-Free Countries

A risk assessment of nine European countries (Austria, Belgium, Germany, Luxembourg, the Netherlands, Slovenia, and Switzerland) considered three main entry pathways for CCHFV:

1. Infected tick vectors;
2. Wildlife reservoirs;
3. Livestock movement.

The overall risk of CCHFV introduction was rated low for most countries but medium for France and Italy, particularly in terms of animal health. In terms of public health risk, Italy was the only country where the risk was classified as medium, while it remained low for all other countries [95]. These findings emphasize the need for continued monitoring and surveillance, particularly in regions with established *Hyalomma* tick populations.

### 4.6. Challenges and Future Predictions for CCHFV Expansion

While *H. marginatum* is spreading into Europe, climatic conditions in some regions may still hinder its overwintering and survival. However, ongoing climate change and habitat change could shift these limitations, making it necessary to continuously update predictive models [93].

Despite its global spread, CCHFV remains more lethal in lower-income regions, such as Africa, where limited healthcare infrastructure contributes to higher mortality rates [90]. Notably, there is a marked difference in CCHFV-associated case fatality rates between regions such as Turkey and Kosovo, with significantly higher lethality reported in Kosovo [96]. This disparity raises important questions regarding regional differences in viral strains, host factors, healthcare infrastructure, or surveillance sensitivity. In contrast, widespread yet cryptic CCHFV circulation has been documented in several African regions, where serological evidence indicates substantial exposure in humans and animals despite a lack of reported clinical cases [89,97]. This phenomenon may be partly attributed to underdiagnosis, particularly given the co-circulation of other hemorrhagic fever viruses that can present with similar clinical syndromes, complicating accurate etiological identification [98,99]. This underscores the need for international collaboration on surveillance, diagnostic improvements, and vaccine research to prevent future outbreaks.

### 4.7. NSDV, ASFV, and the Future of Tick-Borne Viral Infections

In addition to CCHFV, NSDV and ASFV represent significant emerging threats to livestock health, originating in Africa and exhibiting increasing potential for transcontinental spread.

Climate change and increasing animal movement are expected to drive the future spread of NSDV. New diagnostic tools are being developed to improve surveillance and early detection, which will be crucial in mitigating future outbreaks [100]. Ecological niche modeling indicates that within Africa, regions such as Ethiopia, Malawi, and Zimbabwe possess environmental conditions conducive to NSDV transmission, influenced by factors like livestock density, soil moisture, and precipitation. Conversely, areas including the Democratic Republic of Congo, Zambia, and southern Somalia appear less favorable for virus circulation [31]. In addition, many ASFV outbreaks of the disease have been recorded, particularly in this century, and increased pig farming increases the risk of spread and outbreaks [101,102].

As tick-borne viral threats continue to expand, integrated surveillance systems, predictive ecological modeling, and targeted vector control strategies will be essential in preventing the emergence of new zoonotic TBVs and limiting the spread of existing pathogens.

**Table 2:** **Tick species, their genera, and tick-borne viruses associated with geographic distribution in Africa.** This table presents key tick species relevant to human and animal health, categorized by genus. It lists the viruses that each species is known or suspected to transmit, the countries or regions where these tick–virus associations have been detected, and additional notes on vector competence, invasiveness, or ecological relevance. References are included to support the reported associations and geographic data.

Tick Species	Genus	Transmitted Virus(es)	Countries/Regions Detected	Notes	References
***Hyalomma*** **spp.**	*Hyalomma*	CCHFV	Nigeria, Senegal, Ethiopia, Kenya, South Africa, Uganda	Primary vector of CCHFV in Africa.	[49,77]
***H. marginatum***	*Hyalomma*	CCHFV	Spain, France, Mediterranean Basin	Expanding into Europe due to warming climate.	[50]
***H. rufipes***	*Hyalomma*	CCHFV	South Africa, other Sahel/northern African regions	Seasonally active; competent vector.	[103]
***H. truncatum***	*Hyalomma*	CCHFV	Zimbabwe, South Africa	Two larval peaks per year.	[61,74]
***H. impeltatum***	*Hyalomma*	CCHFV	Ethiopia	Experimental transmission confirmed.	[54]
***H. dromedarii***	*Hyalomma*	CCHFV	Northern Africa, Sahel	Common on camels.	[74]
***R. appendiculatus***	*Rhipicephalus*	NSDV, *Theileria parva*, CCHFV	Kenya, East Africa, Uganda	Major vector in East Africa.	[31,104]
***R. microplus***	*Rhipicephalus*	*Babesia* spp., possibly CCHFV	Tanzania, Cameroon, sub-Saharan Africa, Mexico, parts of Asia, South and Central America	Invasive; spreading via livestock trade.	[44,76]
***R. decoloratus***	*Rhipicephalus*	Possibly CCHFV	Kenya, Tanzania	Replaced by *R. microplus* in warmer lowlands.	[60,105]
***A.variegatum***	*Amblyomma*	*Ehrlichia ruminantium*, NSDV	Sub-Saharan Africa	Affects cattle and small ruminants.	[104,106]
***A. hebraeum***	*Amblyomma*	*Ehrlichia ruminantium*, NSDV	South Africa (e.g., Eastern Cape, Limpopo, KwaZulu-Natal)	Peak in summer; key heartwater vector.	[46,47]
***O. moubata***	*Ornithodoros*	ASFV, *Borrelia duttonii*	Eastern and Southern Africa, Malawi	Found in traditional dwellings. Soft tick vector.	[36,55]

## 5. Microbiome Shifts and Vector Competence

**Figure 2:**
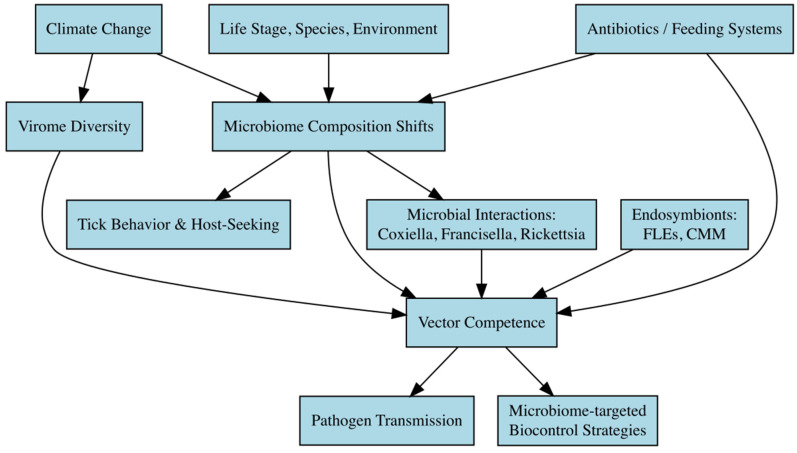
**Conceptual diagram illustrating the pathways through which climate change, microbiome dynamics, and microbial interactions influence tick vector competence and pathogen transmission.** Climate-driven environmental changes affect tick microbiome composition and virome diversity, which in turn modulate tick behavior, host-seeking activity, and vector competence. Intrinsic factors (tick species, life stage) and extrinsic factors (temperature, humidity, feeding system) shape microbial diversity. Key endosymbionts such as *Francisella*-like endosymbionts (FLEs) and *Candidatus Midichloria mitochondrii* (CMM) play central roles in modulating pathogen colonization, tick physiology, and immune responses. Interactions among microbial taxa—competitive (e.g., *Coxiella* vs. *Rickettsia*) or synergistic (e.g., *Francisella* + *Rickettsia*)—further influence pathogen persistence. Antibiotic exposure and artificial feeding systems perturb the microbiome, potentially altering vector competence. These multifactorial interactions ultimately shape the efficiency of pathogen transmission and present opportunities for microbiome-based tick control strategies.

The microbiome of ticks plays a critical role in vector competence, influencing their ability to acquire, maintain, and transmit pathogens. While research has revealed complex interactions between tick-associated bacteria and viruses, significant gaps remain in understanding how climate-driven microbiome shifts affect disease transmission [107]. Figure 2 illustrates these complex relationships.

### 5.1. Climate Change, Microbiome Composition, and Tick Behavior

Increasing temperatures have been shown to alter tick microbiota, potentially impacting tick behavior, host-seeking activity, and consequently, pathogen transmission risk [11]. However, the specific mechanisms through which climate affects tick microbiomes remain largely unknown. Experimental studies are needed to determine how environmental factors influence the composition of tick microbiomes and, in turn, vector competence.

Microbiome composition is highly dynamic and varies across tick life stages, species, and environmental conditions [108]. Notably, studies have shown that *Rhipicephalus* ticks from western and eastern Africa more frequently harbor *Rickettsia* and *Ehrlichia* spp., whereas *Anaplasma* and *Theileria* are more commonly detected in ticks from northern Africa [109]. A study on neotropical ticks in Panama found that microbiome diversity increases from larvae to nymphal stages, with tick species and collection sites being major determinants of microbial variation [110]. Therefore, the results suggest that environmental and tick-specific factors such as species and life stage determine microbial diversity and composition rather than the host blood source. This suggests that both intrinsic (genetic) and extrinsic (environmental) factors shape tick-associated microbial communities [110].

### 5.2. Key Microbial Players and Their Interactions in Ticks

In various African ecosystems, *Coxiella* endosymbionts have emerged as the dominant bacterial group in multiple tick species, while *Francisella* is particularly abundant in *Hyalomma* ticks. Interestingly, *Coxiella* and *Francisella* appear to exhibit a negative correlation, suggesting a competitive interaction. Conversely, a positive association between *Francisella* and *Rickettsia* in *H. rufipes* specimens suggests potential synergistic interactions that may influence pathogen transmission dynamics [111].

Similar trends were observed in ticks from camels in northern Kenya, where *Coxiella* endosymbionts were abundant in *A. gemma* and *R. pulchellus*, while *Francisella* was most present in *Hyalomma* ticks. Both *Coxiella* and *Francisella* were primarily localized in the salivary glands, a critical site for pathogen transmission. Furthermore, high abundances of *Coxiella* and *Pseudomonas* were associated with low *Rickettsia* prevalence, suggesting competitive exclusion between these microbes [112]. These findings highlight that microbial interactions within ticks can modulate vector competence, either enhancing or inhibiting pathogen transmission. In *R. microplus* ticks, the presence of *Theileria* sp. was linked to a significant reduction in microbial diversity, while *H. anatolicum* ticks remained unaffected [113]. This suggests that specific pathogens may selectively disrupt the tick microbiome, potentially benefiting from reduced competition.

### 5.3. Microbiome Diversity and Vector Competence

Tick species capable of transmitting multiple pathogens generally exhibit greater bacterial richness and diversity than those associated with a single pathogen. These differences in bacterial composition support the hypothesis that microbiome diversity plays a key role in shaping vector competence [114]. Moreover, ticks possess defense mechanisms that allow them to maintain pathogens and commensal microbes at levels that do not impair their fitness and development [115]. For instance, *Francisella*-like endosymbionts are essential to *Hyalomma* tick survival and development, playing a role in maintaining tick homeostasis [116].

These interactions highlight the co-evolutionary dynamics between ticks and their microbiota, with potential applications in vector control strategies. Manipulating the tick microbiome could serve as a biocontrol approach, as seen in studies where high levels of *Rickettsia bellii* reduced *Anaplasma marginale* acquisition in *Dermacentor andersoni* ticks, while lower *Francisella* endosymbiont levels correlated with decreased *Francisella novicida* infection [117].

Recent research has highlighted the crucial role of the tick microbiome in modulating the transmission and replication of TBVs. For example, the presence of borrelia in certain tick populations from Germany appears to lower the infection rate with TBEV [118]. The microbiome, composed of both symbiotic and pathogenic microbes, significantly influences tick physiology, immune responses, and vector competence. Notably, *Francisella*-like endosymbionts (FLEs) and *Candidatus Midichloria mitochondrii* (CMM) are dominant components of the tick microbiome, particularly in *Hyalomma* and *Amblyomma* species, which are important vectors of CCHFV and *Rickettsia parkeri*, respectively [116,119]. These endosymbionts can both compete and cooperate with viral pathogens inside the tick, affecting pathogen colonization and transmission. For instance, in *A. maculatum*, CMM supports the replication of *R. parkeri*, while FLEs are reduced in the presence of the pathogen, suggesting a dynamic balance within the tick’s microbial ecosystem [119]. Additionally, endosymbionts contribute to tick homeostasis by synthesizing essential nutrients such as B vitamins, regulating oxidative stress via selenoproteins, and facilitating immune modulation—all of which can indirectly influence viral persistence and transmission capacity [116,119]. Understanding these complex interactions opens up new avenues for controlling TBVs through microbiome-targeted interventions.

### 5.4. The Impact of Climate on Tick Virome Diversity

Climate can directly influence tick immunity and virome composition, thereby affecting vector competence. A study on the invasive tick *Haemaphysalis longicornis* in China found that climate variables exerted a greater influence on virome diversity than other ecological factors, with virome diversity projected to increase in 81.9% of surveyed regions between 2019 and 2030 under current climate scenarios. Specifically, higher temperatures and lower humidity were associated with an increase in vertebrate-associated viral diversity, likely due to changes in host availability [120]. While these findings provide valuable insights, they remain geographically limited. Future work should extend similar high-resolution virome surveys to other biogeographical regions to determine whether analogous climate–virome relationships hold true globally. Longitudinal studies that integrate standardized metagenomic sequencing, detailed host community assessments, and fine-scale climate data will be essential in refining predictive models of tick-borne virus emergence and guiding targeted surveillance and control strategies worldwide.

### 5.5. The Role of Antibiotics and Microbiome Perturbation in Vector Competence

The disruption of the microbiota—for example, through antibiotic treatment—can influence vector competence and alter susceptibility to human pathogens in a range of arthropod vectors [121]. By targeting specific microbial interactions, future research could explore novel biocontrol strategies, reducing tick susceptibility to pathogens and interrupting transmission cycles.

In addition, the influence of antibiotics on the microbiome may also be important in tick feeding research. A comparative feeding study using both an artificial membrane system—supplemented with gentamicin, which is commonly used to stabilize blood in in vitro feeding setups—and live animal hosts demonstrated that both the feeding modality and host species significantly influenced the composition of the tick microbiome [122]. These findings highlight that artificial feeding systems, while valuable for maintaining controlled experimental conditions, may introduce microbiome alterations not representative of natural settings and should therefore be carefully considered when interpreting results related to vector competence or microbial transmission dynamics.

## 6. Immune Mechanisms Driving Vector Competence in Ticks

Ticks rely solely on their innate immune system to defend against viral infections, unlike mammals, which possess both innate and adaptive immunity. Despite their immune defenses, many TBVs have evolved mechanisms to evade, exploit, or suppress tick immunity, allowing for efficient replication, persistence, and transmission to mammalian hosts [123]. Viruses such as CCHFV are mainly maintained in a natural cycle of infection between ticks and domestic or wild animals, and the virus is acquired via a blood meal, where it has to overcome the midgut and salivary gland barriers to establish itself in the tick and be transmitted to the next host [4,124]. Understanding tick immune mechanisms is critical in predicting vector competence and the potential spread of tick-borne viral diseases, especially in the context of climate change.

### 6.1. Innate Immune Pathways in Ticks and Their Role in Vector Competence

Ticks rely exclusively on their innate immune system to defend against pathogens, including bacteria, protozoa, and viruses [125]. Several conserved signaling pathways such as Toll, Janus kinase/signal transducer and activator of transcription (JAK/STAT), and immune deficiency (IMD) contribute to immune responses in arthropods. In ticks, however, the functional roles of these pathways in antiviral defense are not yet fully understood [125,126]. Recent advances in whole-genome sequencing, particularly of *Ixodes* (*I.*) *scapularis*, have identified a range of immunologically relevant genes, including those involved in signaling, redox metabolism, antimicrobial peptide (AMP) production, complement-like responses, and the regulation of apoptosis [127,128,129].

Notably, RNA interference (RNAi) appears to be the most critical immune response, serving as a primary mechanism for controlling viral replication in ticks. Viral double-stranded RNA is processed by Dicer into small interfering RNAs (siRNAs), which guide the RNA-induced silencing complex (RISC) to degrade complementary viral RNA [130]. RNAi core components such as Dicer and Argonaute are present in ticks, and their silencing results in increased viral replication [131]. However, many TBVs have evolved strategies to bypass or exploit RNAi, enhancing their survival and transmission [132]. Notably, tick-borne flaviviruses like *Orthoflavivirus encephalitidis* (Tick-borne encephalitis virus, TBEV) and *Orthoflavivirus langatense* (Langat virus, LGTV) produce subgenomic flavivirus RNAs (sfRNAs) that antagonize the RNAi pathway, promoting viral persistence and enhancing vector competence [131].

Tick cellular immunity includes hemocytes, which mediate phagocytosis, coagulation, and encapsulation [133]. Three hemocyte types have been described: non-phagocytic granulocytes, phagocytic granulocytes, and plasmatocytes [134]. Hemocytes play a role in controlling infections by phagocytosing pathogens [133,135] and supporting antiviral mechanisms such as RNAi [136]. They also generate reactive oxygen species (ROS) during phagocytosis [137], which regulate processes like inflammation, cell death, and tissue repair [138,139]. Interestingly, viral sfRNAs may inhibit apoptosis, as demonstrated for *Orthoflavivirus zikaense* (Zika virus, ZIKV) in mosquitoes, potentially enabling prolonged viral survival in tick cells [140].

While Toll and JAK/STAT pathways appear largely conserved across arthropods [141,142], the IMD pathway in ticks shows divergence. Core IMD components present in *Drosophila melanogaster* are missing in ticks [129], yet alternative IMD-like responses are activated during infections with *Borrelia* or *Anaplasma*, suggesting a tick-specific adaptation of this pathway [143]. Toll and IMD pathways activate NF-κB-like transcription factors, inducing AMP expression [144], which contributes to pathogen inhibition [145].

Finally, tick saliva plays a critical role in vector competence by modulating the vertebrate host immune response, creating a favorable environment for viral replication and transmission [132]. Altogether, the interplay between tick innate immunity, viral immune evasion strategies, and host modulation determines the efficiency of pathogen transmission by ticks.

### 6.2. Tick Immune Interactions with Viruses in a Changing Climate: Insights from ASFV

Compared to TBVs, our understanding of how *Ornithodoros* ticks immunologically interact with ASFV remains limited. However, recent studies suggest that ASFV is capable of modulating the innate immune system of its soft tick vector *O. moubata* to establish persistent infections and enable vertical transmission [146,147]. For example, proteome analysis identified differentially expressed proteins in *O. moubata* cells, associated with metabolism, immune response, reproduction, and pathogen transmission [148]. Moreover, different ASFV strains exhibit varying replication, dissemination, and vertical transmission capabilities across tick species, highlighting the role of species-specific immune responses in vector competence [146].

Climate change is expected to affect the immunity, reproduction, and development of ASFV vector species *O. moubata*, potentially leading to higher feeding rates and expansion to higher latitudes. Rising temperatures may favor these changes, increasing ASFV transmission risks in new areas [149].

Also, rising temperatures might reduce the efficacy of antiviral responses or shift the balance between tolerance and resistance. In several tick species, thermal stress has been linked to increased questing, altered developmental cycles, and changes in reproductive output—all of which may affect virus–host dynamics [150,151,152]. The heat-induced suppression or misregulation of immune pathways could facilitate more efficient ASFV replication and transmission under future climate conditions.

Understanding how ASFV interacts with the immune system of *O. moubata*, and how this relationship is modulated by environmental stressors, is crucial in assessing future transmission risks and developing targeted control strategies.

## 7. Strategies for Monitoring and Mitigating Emerging Tick-Borne Viral Threats in Africa

Emerging tick-borne viral threats, particularly CCHFV, pose a growing risk in Africa, exacerbated by climate change, expanding tick populations, and evolving vector competence. Addressing these challenges requires a multifaceted strategy that includes enhanced surveillance, sustainable control methods, vaccine development, and stronger healthcare infrastructure.

### 7.1. Strengthening Surveillance and One Health Approaches

A comprehensive surveillance system is essential in managing tick-borne viral threats in Africa. The molecular identification of tick species, their distribution, and their pathogen interactions can significantly improve risk assessments and inform targeted interventions [153]. A sustainable One Health strategy, integrating human, animal, and environmental health, is necessary to prevent the spread of TBVs like CCHFV. This approach includes early warning systems, risk reduction strategies, and international collaboration [154,155].

Climate change is altering vector-borne disease patterns, making it critical for African countries to adapt to climate-related health risks. Understanding weather and climate trends and incorporating this knowledge into vector control strategies can enhance disease mitigation efforts [71].

### 7.2. Developing Alternative Tick Control Strategies

Traditional acaricide-based tick control methods face significant challenges, including environmental burdens and the emergence of acaricide-resistant tick populations. Anti-tick vaccines present a promising alternative, particularly in sub-Saharan Africa, where ticks negatively impact livestock and human health. Several vaccine candidates are currently in development, offering a sustainable and environmentally friendly method to reduce tick-borne pathogen transmission [156]. Targeting tick immunodeficiency mechanisms represents another potential avenue for the development of anti-tick vaccines, reducing the reliance on chemical acaricides [157].

Additionally, innovative vaccine research is exploring the potential of targeting keystone bacteria in the tick microbiome, which may alter vector competence and pathogen transmission [158,159]. This microbiome-focused approach could disrupt pathogen survival within ticks, limiting their ability to transmit viral threats to humans and animals.

### 7.3. Antiviral Control Strategies

In addition to the application of anti-tick treatments preventing tick infestations, the development of effective vaccines against TBVs represents a crucial component of public health strategies. TBEV is a well-known example for which a safe and effective vaccine is already in use in several endemic regions [160]. In contrast, despite significant research progress, there is currently no licensed vaccine for CCHFV. In particular, the genetic variability of CCHFV strains makes the development of a broad-spectrum vaccine challenging [161]. Only in recent years has research on CCHFV intensified, as its expanding distribution and the recognition of its epidemic potential have made this virus a focus in global public health efforts [162].

Besides the vaccine development efforts in CCHFV, increasing attention has been paid to immunoprophylaxis against other tick-borne viruses, such as ASFV and NSDV, which affect livestock with severe lethality. In the case of ASFV, although several live attenuated and subunit vaccine candidates have shown partial efficacy in experimental models, no licensed vaccine is currently available [34,163]. As with ASFV, there is currently no commercially available vaccine for NSDV. Although experimental vaccines have been deployed in endemic regions and have been shown to confer protective immunity in small ruminants, concerns regarding biosafety and reversion to virulence, as well as genetic and thermal stability, persist, and ongoing research efforts aim to develop a safer and more stable vaccine candidate [30,164,165,166]. These limitations underscore the need for advanced vaccine platforms that ensure both efficacy and safety under field conditions.

### 7.4. Enhancing Healthcare Infrastructure and International Cooperation

The SARS-CoV-2 pandemic underscored the urgent need for stronger health systems in Africa, particularly to tackle emerging infectious diseases like tick-borne viral infections. A strengthened health concept must include localized improvements in healthcare infrastructure and international partnerships to bolster Africa’s response to vector-borne disease threats [167].

CCHF represents a significant burden for Africa, making prevention, improved diagnostics, and effective treatments essential. Stronger healthcare systems, enhanced cooperation, and investments in medical research can improve early detection, outbreak response, and patient outcomes [168].

## 8. Conclusions

The rapid expansion of TBVs, particularly CCHFV, underscores the urgent need for global cooperation in surveillance, mitigation, and disease prevention strategies. Climate change remains a significant driver of tick distribution shifts, altering vector behavior and competence and increasing the risk of viral spillover in both endemic and previously unaffected regions. Africa, in particular, is witnessing an intensification of tick-borne disease burden, as expanding tick habitats—driven by rising temperatures, land-use changes, and ecological disruption—facilitate greater human and animal exposure.

Key conclusions from the current body of literature point to a convergence of environmental, biological, and socio-economic drivers fueling the emergence of TBVs. Traditional tick control methods, such as acaricides, are proving increasingly ineffective due to rising resistance and environmental impact. At the same time, the cross-border movement of livestock, migratory birds, and evolving land-use practices are accelerating the geographic spread of ticks and associated viruses.

Beyond Africa, the spread of *Hyalomma* ticks and CCHFV into parts of Europe and Asia signals a growing global threat, necessitating enhanced predictive modeling and regional preparedness. This includes establishing real-time entomological and clinical surveillance networks, harmonized cross-border reporting systems, improved diagnostic laboratory capacities, and coordinated outbreak response frameworks.

However, critical knowledge gaps remain. There is still no licensed vaccine for major TBVs such as CCHFV, NSDV, and ASFV, despite their growing zoonotic and economic impact. The long-term effects of climate change on vector–pathogen–host interactions remain poorly understood, limiting the accuracy of disease forecasting models. Furthermore, research into microbiome-driven vector competence and immune modulation is still in the early stages, and the safety and stability of experimental vaccines under field conditions require further evaluation.

To address these challenges, the following actionable recommendations are proposed:

- Strengthening surveillance by scaling up molecular tick identification and TBV monitoring across diverse ecological zones;
- Adopting a One Health approach to integrating human, animal, and environmental health responses in TBV control strategies;
- Investing in vaccine development and prioritizing scalable and broadly effective candidates for TBVs, particularly for high-risk livestock populations;
- Promoting innovative control strategies, such as anti-tick vaccines and microbiome-targeted interventions, as sustainable alternatives to chemical acaricides;
- Enhancing healthcare infrastructure and diagnostics, especially in high-burden, low-resource settings, to improve outbreak response and clinical outcomes;
- Facilitating international collaboration, data sharing, and standardized protocols to track and manage the transboundary spread of TBVs.

## Data Availability

No new data were created or analyzed in this study. Data sharing is not applicable to this article.

## Abbreviations

The following abbreviations are used in this manuscript:

*A.*	*Amblyomma*
AMP	antimicrobial peptide
ASFV	African swine fever virus
CCHF	Crimean–Congo hemorrhagic fever
CCHFV	*Orthonairovirus haemorrhagiae* (Crimean–Congo hemorrhagic fever virus)
CMM	*Candidatus Midichloria mitochondrii*
*H.*	*Hyalomma*
*I.*	*Ixodes*
IMD	immune deficiency
JAK/STAT	Janus kinase/signal transducer and activator of transcription
MDPI	Multidisciplinary Digital Publishing Institute
NSDV	*Orthonairovirus nairobiense* (Nairobi sheep disease orthonairovirus)
*O.*	*Ornithodoros*
*R.*	*Rhipicephalus*
RNAi	RNA interference
ROS	reactive oxygen species
sfRNAs	subgenomic flavivirus RNAs
siRNAs	small interfering RNAs
TBV	Tick-borne virus
